# Impact of Shyness on Self-Esteem: The Mediating Effect of Self-Presentation

**DOI:** 10.3390/ijerph19010230

**Published:** 2021-12-26

**Authors:** Adrianna Bober, Ewa Gajewska, Anna Czaprowska, Agata Hiacynta Świątek, Małgorzata Szcześniak

**Affiliations:** Institute of Psychology, University of Szczecin, 70-017 Szczecin, Poland; adrianna.bober@usz.edu.pl (A.B.); psycholog.ewagajewska@gmail.com (E.G.); czapranna1a@gmail.com (A.C.); hiacynta.swiatek@wp.pl (A.H.Ś.)

**Keywords:** shyness, self-esteem, self-presentation, self-promotion, self-deprecation, mediation

## Abstract

Background: Although the relationship between shyness and self-esteem is well described in the psychological literature, far less is known about the potential mechanisms that underlie this association. The main goal of the current work is to verify whether self-presentation acts as a mediating variable between both constructs. Methods: The study was carried out among 198 adults. The Revised Cheek and Buss Shyness Scale, the Rosenberg Self-Esteem Scale, and the Self-Presentation Style Questionnaire were applied. Results: A large and positive correlation coefficient was observed between the following variables: (1) self-esteem/self-promotion; (2) shyness/self-deprecation. All other variables correlated negatively: (1) shyness/self-esteem; (2) shyness/self-promotion; (3) self-esteem/self-deprecation; (4) self-promotion/self-deprecation. Moreover, both self-promotion and self-deprecation acted as mediators between life satisfaction and self-esteem. Conclusion: The outcomes of the present study show a new mediating aspect for the direct relationship between shyness and self-esteem in the form of two styles of self-presentation. The results indicate that the tendency of shy people to avoid others can have a lower effect on their overall sense of self-esteem when they try to present themselves in a clearly favorable light. By contrast, shyness may have a stronger impact on their sense of self-worth when they present themselves as helpless, unsure, and incompetent.

## 1. Introduction

The research problem of the article is the relationship between shyness, self-esteem, and styles of self-presentation. Although the concept of shyness is difficult to describe [1], there is some agreement that shyness is a tendency to avoid people [2] and feel inhibition, tension, unpleasant emotions, or discomfort in social interactions [3]. It affects many fields of the shy person’s functioning [4,5].

According to different researchers, shyness may be considered both as an emotional state and a personality trait [2,4,5]. A state of situational shyness denotes an emotional and cognitive response to some specific, novel, temporary, and threatening social circumstances that everyone can experience in the presence of others [5,6,7]. Cheek and Buss [2] consider shyness as a stable construct and one of the most widespread and common personality traits. Shyness can be a source of unpleasant emotions or discomfort (frequent embarrassment or fear). It can also make it difficult to experience positive emotions when it is the source of a sense of loss (e.g., “I didn’t try it, even though I had the opportunity”). Finally, it can affect the way a shy person is perceived by the environment (e.g., “He looks worse during a job interview”). It can be assumed that when shyness is strongly increased, it reduces the quality of life of the person it concerns. Indeed, a study on a Chinese group of students shows that shyness is negatively associated with life satisfaction [8].

Extensive research shows that a reduced self-esteem is one of the crucial factors associated with shyness [9]. Self-esteem is a complex and multidimensional concept that refers to a person’s attitude toward oneself [10]. Blascovich and Tomaka [11] define self-esteem as “the sum of evaluations across salient attributes of one’s (…) personality” (p. 115). In psychology research, self-esteem is considered to be a construct consisting of two different dimensions [12]. The competence facet expresses the degree to which individuals perceive themselves as skilled and successful. The worth dimension denotes the degree to which people experience themselves as valuable. Self-esteem has been viewed as a motive [12,13,14], buffer [12,15,16], and outcome variable [12,15,17]. Assuming the third perspective, some researchers have found that self-esteem may depend on personality traits [18,19], interpersonal factors such as social feedback, approval or disapproval [20,21,22], the relationships with parents and significant others [23,24], personal achievement [25] or career success [26], and shyness [27].

The last of these variables is of interest for at least two reasons. First, shyness occurs in a considerable number of people at some point in their lives [28,29,30] and, second, shyness is a social phenomenon and, as such, may affect the sense of self-esteem [31]. Empirical findings display that shyness correlates negatively with self-esteem among preschoolers [32], school children [33,34], adolescents [35,36], as well as younger [37,38,39] and older adults [31]. In other words, people who are shy typically tend to have a lower perception of self-worth [40] and have a lower [38,41] or negatively unrealistic [40] self-evaluation of themselves and their abilities. Based on the above studies, we hypothesized that:

**Hypothesis** **1** **(H1).** *Shyness is negatively correlated with self-esteem.*

It may seem that Hypothesis 1 (H1), considered in isolation from the other hypotheses, adds little new knowledge to the existing literature. However, some research on shyness cited as the foundation for this hypothesis dates to the 1980s. The authors of the studies at that time could not predict to what extent the socio-cultural conditions would change, i.e., that self-image, the attitude toward oneself, and self-esteem (potentially important for shyness) are now built in a slightly different way, using different tools and experiences (social media), and that this process is attended by, inter alia, people unknown to the subject (observers and subscribers). All of the participants in this survey are regular social media users. Saunders and Chester [1] write about the role of social media in the functioning of shy people. They can be attractive because self-image can be freely created and information about yourself can be better controlled, while strengthening the real isolation of shy people. At the same time, they are indicated as a disinhibiting medium and help to train social skills. Therefore, it seems justified to verify the seemingly known relationship of shyness with self-esteem.

There are relatively few studies reporting a direct relationship between shyness and self-presentation. According to Schlenker and Leary [42], self-presentation is a process by which people “attempt to control images of the self before real or imagined audiences” (p. 643). People adopt self-presentation styles to gain a certain outcome or to induce an evaluation by others [43]. Different types of self-presentation are used to affect other peoples’ impressions of the self [44]. Acquisitive self-presentation occurs when people try to accentuate pleasing aspects of themselves to receive others’ approval [45]. Conversely, protective self-presentation takes place when people want to avoid disapproval. Assertive self-presentation involves behaviors directed at focusing attention on an identity [44]. In contrast, defensive self-presentation denotes actions intended for defending an identity. Attributive self-presentation refers to claiming positive, kind, desirable characteristics [43,46]. On the other hand, repudiative self-presentation implies denying negative, undesirable characteristics. Finally, self-promotion is related to the enhancement of the self [47] through emphasizing competences, strengths, and talents [48]. On the contrary, self-deprecation reflects being incompetent, insecure, and helpless [49].

In the context of shyness, researchers suggest that people with higher levels of social fear are more convinced of their inability to make a favorable impact on others. At the same time, they are highly motivated to create a desired image of themselves. Szmajke [50] explains that shy people tend to use defensive styles of self-presentation, aimed more at avoiding negative impressions than actively striving to exert a positive one. In other words, they do not aim to demonstrate their strengths, instead preferring to not disclose unfavorable information about themselves. Along the lines of the previous studies, we hypothesized that:

**Hypothesis** **2** **(H2).** *Shyness is negatively correlated with self-promotion, and positively associated with self-deprecation.*

Although there is some empirical evidence that self-presentation is not related to self-esteem [51,52], Hermann and Arkin [43] suggest that individual differences in adapting various self-presentation tactics are related to people’s self-esteem. Given that people aspire to sustain, defend, and strengthen their self-esteem [1], different self-presentation styles may contribute [53] positively or negatively to the realization of this goal. For example, Leary [46] observes that a lack of success in self-presenting may lower one’s self-esteem, leading to an increase in negative emotions. Conversely, an achievement in self-presentation may raise self-esteem and elicit positive emotions. Other studies [11] show that an acquisitive strategy of self-presentation characterizes people who demonstrate a higher self-esteem, while a protective self-presentation distinguishes people who declare a lower self-esteem. In turn, Hewitt et al. [54] observed that the perfectionistic tactic of self-presentation, one that consists of creating an image of flawlessness to others, correlates negatively with self-esteem. Based on a literature review, we postulated that:

**Hypothesis** **3** **(H3).** *Self-promotion correlates positively with self-esteem, and self-deprecation is negatively associated with self-esteem.*

Although the relationship between shyness and self-esteem is well described in the psychological literature, far less is known about the potential mechanisms that underlie this association. Hence, the main goal of the current work is to verify whether self-presentation could act as a mediating variable between both constructs. There are several reasons for selecting self-presentation as a mediator from a range of different options. Specifically, shyness is “an extreme concern with self-presentation” (p. 2650) [23]. Moreover, self-presentation, besides its important function of “pleasing the audience,” plays an equally essential role of self-construction. According to Baumeister [55], people not only try to look good in the eyes of others or impress them, but also seek to act according to their own ideals and values. In doing so, their self-presentation motivates people to “create, maintain, and modify” (p. 3) [56] their image, and this may be important in influencing their self-esteem. Moreover, in some other studies, self-presentation mediated the relationship between perfectionistic concerns and subjective well-being [57], and life satisfaction and self-esteem [58]. Based on the presented insights, we hypothesized that:

**Hypothesis** **4** **(H4).** *Self-promotion and self-deprecation act as mediators between shyness and self-esteem.*

Overall, our main purpose is to check whether feeling inhibited and tense in social interactions has a greater or lesser effect on one’s general sense of value when they enhance aspects of the self or disparage one’s own abilities.

## 2. Materials and Methods

### 2.1. Participants and Data Collection

The study was carried out among 198 Polish adults—157 women (79.3%) and 41 men (20.7%) aged 15 to 70 years (*M* = 26.66; *SD* = 8.97). The data were collected through a set of self-reported questionnaires with the use of the internet (e.g., Facebook). In the present work, we used a cross-sectional study and a convenience sample as such a methodology involves less time and fewer resources while being simple to apply [59]. All of the participants were given an accurate explanation of the goals of the study and about the confidentiality of the data, and were required to complete a web-based informed consent form. After providing their agreement, the respondents were asked to complete all of the scales. The topic and the procedure were accepted by the Institutional Bioethics Committee (KB 17/2021). The study was completed in line with the ethical recommendations present in the Declaration of Helsinki.

### 2.2. Measurements

Revised Cheek and Buss Shyness Scale (RCBS). A self-report measure, developed by Cheek and Buss [24] and adapted into Polish by Kwiatkowska et al. [60], which is used to assess shyness understood as a personality trait. It is conceived as a unidimensional questionnaire and consists of 13 items (e.g., “I feel tense when I’m with people I don’t know well”; “I feel inhibited in social situations”). The participant assesses the extent to which each of the statements applies to his or her life. The answers are given using a five-point Likert scale (from 1—“very uncharacteristic or untrue”—to 7—“very characteristic or true”). Scores range from 13 to 65, with higher results reflecting greater levels of shyness. In the current study, the RCBS presented very good reliability, with a Cronbach’s alpha reliability coefficient of α = 0.89.

Rosenberg Self-Esteem Scale (RSES). A self-report research instrument, created by Rosenberg [61] and adapted into Polish by Łaguna et al. [62], which is used to measure one’s global perception of worthiness. This unidimensional tool consists of 10 items. Five of them are positively worded (e.g., “On the whole, I am satisfied with myself”) and five are negatively worded (e.g., “I feel I do not have much to be proud of”). The participants declare on a four-point scale (from 1—“strongly agree”—to 4—“strongly disagree”) to what degree they agree with each of the items. The total score for the RSES is created by summing the values of the items, with higher scores denoting higher self-esteem. The present study had an excellent value of Cronbach’s alpha reliability coefficient (α = 0.90).

Self-Presentation Style Questionnaire (SSQ). A two-factor questionnaire, developed by Wojciszke [63], which estimates styles of self-presentation, understood as intended behavior aimed at altering the way other people regard themselves and inducing the desired impact on others. These styles are generally consistent with what people really think about themselves. The questionnaire consists of 30 items forming 2 factors of 15 items each that make up the distinguished styles of self-presentation: self-promotion—presenting oneself in a clearly favorable light as a competent person equipped with knowledge and numerous skills (e.g., “I emphasize my own merits”); self-deprecation—presenting oneself as a modest, helpless, unsure, and incompetent person burdened with faults (e.g., “I belittle the importance of my achievements”). The participants respond to all statements using a 5-point scale (from 1—“never”—to 5—“very often”). The present study had high α values of Cronbach’s alpha reliability coefficients for both styles of self-presentation: self-promotion (α = 0.87) and self-deprecation (α = 0.84).

### 2.3. Statistical Analysis

All statistical analyses were completed using IBM SPSS Statistics 25.0 with a 95% confidence level. Because the set of questionnaires was administered via the internet, no missing data were detected among the considered observations. Moreover, pre-analysis screening was performed, using Mahalanobis and Cook’s distance indicators to detect possible outliers. To assess the extent of correlation between all of the variables, the Pearson correlation coefficients were calculated.

The verification of H3, concerning the analysis of the two simple mediation models, was conducted using a bootstrapping-based technique with 5000 resamples and PROCESS macro 3.4 (Heinrich-Heine-Universität, Düsseldorf, Germany) (Model 4) [64]. The analyses displayed statistically significant values of standardized regression coefficients for both mediation models, where shyness was considered as the independent variable (X), self-esteem as the dependent variable (Y), with self-promotion in model 1 and self-deprecation in model 2 as the mediators (M).

## 3. Results

### 3.1. Descriptive Statistics

Table 1 shows the descriptive statistics of the mean, standard deviation, possible range of variables, skewness, and kurtosis for shyness, self-esteem, and both dimensions of self-presentation. Although there is no unequivocal cutoff to designate an unacceptable level of skewness and kurtosis, we assumed a conservative approach to both indices of lower than +/− 1. No variables exceeded this cutoff.

### 3.2. Correlations

The analysis carried out with the use of Pearson’s *r* coefficient (Table 2) showed statistically significant (*p* < 0.001) correlations between shyness, self-esteem, and both dimensions of self-presentation. In accordance with H1, shyness correlated negatively with self-esteem (*r* = −0.50). Secondly, H2 was confirmed as shyness was associated negatively with self-promotion (*r* = −0.52) and positively with self-deprecation (*r* = 0.54). Likewise, self-esteem was positively correlated with self-promotion (*r* = 0.51) and negatively with self-deprecation (*r* = −0.56).

On the bases of the correlational results obtained in the present study, it can be acknowledged that linear relationships between variables were large and medium. Shy people showed a lower self-esteem and self-presentation, and displayed higher self-deprecation. Moreover, individuals with higher levels of self-esteem revealed a higher self-presentation and lower self-deprecation.

### 3.3. Mediation Analyses

In terms of H4, the two simple mediation analyses revealed a statistically significant effect related to both self-presentation dimensions on the relationship between shyness and self-esteem. The strength of the total indirect effect was slightly higher for the model with self-deprecation as a mediator (total indirect effect = −0.13) than for self-promotion (total indirect effect = −0.10).

**Figure 1:**
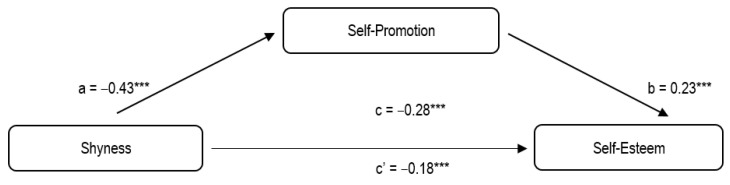
Results of mediation analysis of self-promotion in the relationship between shyness and self-esteem. *** *p* < 0.001.

More specifically, for the first model (Figure 1), statistically significant (*p* < 0.001) values of the regression coefficients were observed between shyness and self-promotion—path a (β = −0.43)—, and between the style of self-promotion and self-esteem—path b (β = 0.23). After including self-promotion as the mediator, the original value of the regression coefficient decreased from β = −0.28 (c) to β = −0.18 (c’), remaining with the same significance level.

**Figure 2:**
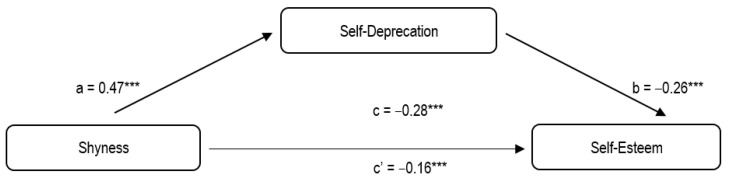
Results of mediation analysis of self-deprecation in the relationship between shyness and self-esteem. *** *p* < 0.001.

Likewise, in the second model (Figure 2), statistically significant (*p* < 0.001) non-standardized coefficients were found between shyness and self-deprecation—path a (β = 0.47)—and self-deprecation and self-esteem—path b (β = −0.26). Furthermore, according to the higher value of the total indirect effect for the second model, the initial value of the regression between the independent and the dependent variable (β = −0.28) decreased to a greater extent than in the case of the first analyzed model (β = −0.16).

In both cases, the confidence interval did not include the value “0”, which confirmed their statistical significance. For the first model, it was B (SE) = −0.1013 (0.0253), 95% CI [−0.1546; −0.0567]. For the second model it was B (SE) = −0.1262 (0.0249), 95% CI [−0.1769; −0.0781].

## 4. Discussion

The present work supported the following hypotheses: shyness is negatively related to self-esteem (Hypothesis 1 (H1)) and self-promotion (Hypothesis 2 (H2)); shyness is positively associated with self-deprecation (Hypothesis 2 (H2)); self-promotion correlates positively with self-esteem, and self-deprecation is negatively associated with self-esteem (Hypothesis 3 (H3)); self-promotion and self-deprecation act as mediators between shyness and self-esteem (Hypothesis 4 (H4)).

First, the inverse correlation between shyness and self-esteem was in line with several other studies, which provided evidence that shy individuals are typically inclined to have a low opinion and to maintain negative self-images of themselves [19]. Although the cross-sectional character of the present study did not allow us to assume that shyness leads to self-esteem, Brown et al. [65,66] suggested that self-esteem depends on interpersonal and temperamental factors. In fact, the self develops in a relational context of being with other people, perceiving their acceptance, and experiencing different social activities [67,68]. Moreover, shyness has a strong genetic component [69], which predisposes people to feel awkwardness on exposure to unknown people or novel situations [70,71]. Carducci and Golant [72] observed that shyness affects all aspects of people’s existence, influencing their behaviors, thoughts, and emotions. The classic view of well-being consists of three components [73], which include the level of life satisfaction, positive feelings, and no negative feelings. In this context, shyness is an important factor in building well-being, for example, shyness decreases self-efficacy [29,74] and self-confidence [41]. Shy individuals reported self-blame [74], anger [74], and emotion-focused coping strategies [75]. Thereby, it is understandable that higher levels of shyness may contribute to the weakening of self-esteem.

In respect to shyness and both dimensions of self-presentation, Hypothesis 2 (H2) also found its confirmation. For example, Arkin [45] argued that shy people regulate their images of self, avoiding the disapproval of others rather than gaining their approval. Other studies show that shyness is related to the perception of one’s own inadequacy [76], the negative judgment of the self [77] and high personal responsibility for any lack of success [24]. These outcomes confirmed the premises of the self-presentational theory of shyness according to which people aim to control images of the self in a social context to depict themselves in the best way for them or the way most suitable to the situation [78]. In conformity with theories of social cognition, shy people presented themselves in a biased or incorrect way [79] to reduce their uncomfortable feelings in the presence of other people [42]. They are often afraid of committing a mistake or failing in social situations [80]. Snyder et al. [80] observed that shy individuals may use strategies of self-handicapping. People who employ such self-presentation tactics do so to excuse for their poor performance and failure. Other authors [45,81] showed that shy people tend to employ a protective self-presentational style since they prefer to avoid social disapproval rather than to achieve others’ approval. Mandal and Wierzchoń [82] revealed that shy individuals are inclined to adopt self-deprecation more often than their less shy counterparts. Simultaneously, non-shy people use self-promotion more frequently than their shy peers. Therefore, our findings suggested that shy people tend to self-deprecate themselves to avoid the negative feelings related to the potential for defeat.

Regarding the dimensions of self-presentation and self-esteem (Hypothesis 3 (H3)), consistent with other studies, self-promotion correlated positively with self-esteem, and self-deprecation was negatively associated with self-esteem. In fact, it has been found [83] that people who present themselves as reserved, silent, and withdrawn, are prone to demonstrate lower self-esteem. They are also less concerned with self-promotion. Similarly, McGregor and Jordan [84] observed that promotion/approach motivation positively correlated with self-esteem. In contrast, prevention/avoidance is associated negatively with self-esteem. According to Speer [85], self-deprecation manifests negative self-regard and reflects low self-esteem. Portraying the self as less capable, ‘not good enough’ [86], and ‘worse-than-average’ [87] indicates a negative sense of self. Research performed in the context of social media corroborates the concept that authentic forms of self-presentation positively associate with increased levels of reported self-esteem, while inauthentic forms of self-presentation correlate with a low self-esteem [88,89]. According to Archibald and Chen [90], such relationships arise from psychological compatibility and a desire to avoid or reduce cognitive discrepancy. Therefore, more negative self-presentation coincides with a lower self-esteem. Moreover, Gonzales and Hancock [91] demonstrated that selective self-presentation on social media, often limited to positive details, may increase self-esteem since information posted there is usually carefully chosen and positive. Unfortunately, adhering to the self-depressive style of presentation may seem safer than the risks that come with self-promotion. Crowden [92] writes that shyness and worry are related. Shy people are more likely to worry and fear social evaluation. Potential failure may seem more inhibiting to a shy person than the benefits of emphasizing strengths. The role of individual mental resilience as a potential resource in changing the strategy of self-presentation seems interesting and it would be worth considering in future studies.

Finally, the mediatory effect of self-promotion and self-deprecation illustrated that the straightforward relationship between shyness and self-esteem may be altered by these two self-presentation tactics. Specifically, it can be tentatively implied that a tendency to evade social interactions can induce a lower or higher self-esteem depending on the character of the self-presentation styles, which can motivate individuals to modify their image. In fact, it is widely recognized that self-presentation styles are important for shy people [23]. In the case of self-promotion that consists of emphasizing one’s own merits and talents, shy people who use this strategy may present themselves as more competent and knowledgeable. Consequently, such a style may lead to improving their self-esteem. Nevertheless, if they use self-deprecative tactics and belittle themselves or their achievements, such an approach may negatively affect their level of self-esteem.

## 5. Limitations

The main concern with this study was that it depends on self-report measures. In the future, it would be recommended to use other methodological approaches to ascertain a greater objectivity and confirm the findings. The sample was also predominantly restricted to women and had an uneven age distribution, which did not allow the generalization of the results. Hence, subsequent studies should include a more balanced sample. Finally, the study had a cross-sectional character and, therefore, we could not imply the existence of causal associations among shyness, self-esteem, and both styles of self-presentation. Thus, longitudinal and experimental studies should be conducted in the future to validate the present findings.

Although shyness itself has been of interest to psychologists for many years, the current conditions for establishing and maintaining social relationships have changed. Therefore, it seems worthwhile to update our knowledge and expand the research on this phenomenon with other qualitative and quantitative research.

## 6. Conclusions and Implications

The outcomes of the current study confirmed all hypotheses and illustrated a new mediating aspect as a direct relationship between shyness and self-esteem in the form of two styles of self-presentation. Our results provided the initial insight that the tendency of shy people to avoid others can have a lower effect on their overall sense of self-esteem when they try to present themselves in a clearly favorable light. By contrast, shyness may have a stronger impact on their self-worth when they present themselves as helpless, unsure, and incompetent.

It remains for practicing psychologists to consider the implications. Presumably, modeling behavior at work with overly shy people should aim at them adopting self-promotional strategies. This strategy, which at first causes cognitive dissonance [93] because of measures that reduce internal tension, may lead to changes in beliefs. Shy people will strive to change their opinion, in this case, their beliefs about themselves, in such a way as to match their new behavior or words. Moreover, those who learn to emphasize their strengths through self-promotion may not only start to present themselves more favorably in life situations that are important to them (e.g., during a job interview), but may also start to see their value and value themselves more. This could allow a shy person to seize opportunities more boldly and engage in new relationships or challenges; thus, leading to an improved self-esteem and overall well-being. That is why it is important for shy children and adolescents to build trust and conviction about their own competences. It can be conducted through encouraging them to talk about their strengths. In this way, they will notice their qualities and feel better about themselves.

Moreover, some other factors, not included in the study, could have an important mediating role between shyness and self-esteem. Among the various variables, there can be considered those that are linked with shyness as well as self-esteem. Their use could provide new information on the possible mechanisms that underlie this relationship: hope, creativity, or sensitivity, to name just a few examples.

## Figures and Tables

**Table 1:** Descriptive statistics for shyness, self-esteem, self-promotion, and self-deprecation (N = 198).

Variable	M	SD Min/Max	Skewness	Kurtosis
Shyness	39.35	11.64 13 65	−0.110	−0.530
Self-Esteem	26.99	6.66 10 40	−0.139	−0.473
Self-Promotion	41.62	9.85 15 71	0.103	0.428
Self-Deprecation	44.20	10.06 15 72	0.153	0.390

**Table 2:** Pearson correlation coefficients between the analyzed variables.

	Shyness	Self-Esteem	Self-Promotion	Self-Deprecation
Shyness	1			
Self-Esteem	−0.50 ***	1		
Self-Promotion	−0.52 ***	0.51 ***	1	
Self-Deprecation	0.54 ***	−0.56 ***	−0.38 ***	1

*** *p* < 0.001.

## Data Availability

The datasets used during the current study are available from the corresponding author.
